# Prevalence, Characteristics, and Outcomes of Emergency Department Discharge Among Patients With Sepsis

**DOI:** 10.1001/jamanetworkopen.2021.47882

**Published:** 2022-02-10

**Authors:** Ithan D. Peltan, Sierra R. McLean, Emily Murnin, Allison M. Butler, Emily L. Wilson, Matthew H. Samore, Catherine L. Hough, Nathan C. Dean, Joseph R. Bledsoe, Samuel M. Brown

**Affiliations:** 1 Division of Pulmonary and Critical Care Medicine, Department of Medicine, Intermountain Medical Center, Murray, Utah; 2 Division of Pulmonary and Critical Care Medicine, Department of Internal Medicine, University of Utah School of Medicine, Salt Lake City; 3 University of Utah School of Medicine, Salt Lake City; 4 Department of Physical Medicine and Rehabilitation, University of North Carolina School of Medicine, Chapel Hill; 5 Department of Medicine, University of Wisconsin School of Medicine, Madison; 6 Statistical Data Center, Intermountain Healthcare, Murray, Utah; 7 Divisions of Epidemiology and Infectious Disease, Department of Medicine, University of Utah School of Medicine, Salt Lake City; 8 Division of Pulmonary, Critical Care, and Sleep Medicine, Department of Medicine, Oregon Health and Sciences University, Portland; 9 Department of Emergency Medicine, Intermountain Medical Center, Murray, Utah; 10 Department of Emergency Medicine, Stanford University, Palo Alto, California

## Abstract

**Importance:**

Sepsis guidelines and research have focused on patients with sepsis who are admitted to the hospital, but the scope and implications of sepsis that is managed in an outpatient setting are largely unknown.

**Objective:**

To identify the prevalence, risk factors, practice variation, and outcomes for discharge to outpatient management of sepsis among patients presenting to the emergency department (ED).

**Design, Setting, and Participants:**

This cohort study was conducted at the EDs of 4 Utah hospitals, and data extraction and analysis were performed from 2017 to 2021. Participants were adult ED patients who presented to a participating ED from July 1, 2013, to December 31, 2016, and met sepsis criteria before departing the ED alive and not receiving hospice care.

**Exposures:**

Patient demographic and clinical characteristics, health system parameters, and ED attending physician.

**Main Outcomes and Measures:**

Information on ED disposition was obtained from electronic medical records, and 30-day mortality data were acquired from Utah state death records and the US Social Security Death Index. Factors associated with ED discharge rather than hospital admission were identified using penalized logistic regression. Variation in ED discharge rates between physicians was estimated after adjustment for potential confounders using generalized linear mixed models. Inverse probability of treatment weighting was used in the primary analysis to assess the noninferiority of outpatient management for 30-day mortality (noninferiority margin of 1.5%) while adjusting for multiple potential confounders.

**Results:**

Among 12 333 ED patients with sepsis (median [IQR] age, 62 [47-76] years; 7017 women [56.9%]) who were analyzed in the study, 1985 (16.1%) were discharged from the ED. After penalized regression, factors associated with ED discharge included age (adjusted odds ratio [aOR], 0.90 per 10-y increase; 95% CI, 0.87-0.93), arrival to ED by ambulance (aOR, 0.61; 95% CI, 0.52-0.71), organ failure severity (aOR, 0.58 per 1-point increase in the Sequential Organ Failure Assessment score; 95% CI, 0.54-0.60), and urinary tract (aOR, 4.56 [95% CI, 3.91-5.31] vs pneumonia), intra-abdominal (aOR, 0.51 [95% CI, 0.39-0.65] vs pneumonia), skin (aOR, 1.40 [95% CI, 1.14-1.72] vs pneumonia) or other source of infection (aOR, 1.67 [95% CI, 1.40-1.97] vs pneumonia). Among 89 ED attending physicians, adjusted ED discharge probability varied significantly (likelihood ratio test, *P* < .001), ranging from 8% to 40% for an average patient. The unadjusted 30-day mortality was lower in discharged patients than admitted patients (0.9% vs 8.3%; *P* < .001), and their adjusted 30-day mortality was noninferior (propensity-adjusted odds ratio, 0.21 [95% CI, 0.09-0.48]; adjusted risk difference, 5.8% [95% CI, 5.1%-6.5%]; *P* < .001). Alternative confounder adjustment strategies yielded odds ratios that ranged from 0.21 to 0.42.

**Conclusions and Relevance:**

In this cohort study, discharge to outpatient treatment of patients who met sepsis criteria in the ED was more common than previously recognized and varied substantially between ED physicians, but it was not associated with higher mortality compared with hospital admission. Systematic, evidence-based strategies to optimize the triage of ED patients with sepsis are needed.

## Key Points

**Question:**

What are the prevalence, characteristics, and outcomes of discharge to outpatient treatment of emergency department (ED) patients with sepsis?

**Findings:**

In this cohort study of 12 333 adult ED patients who met sepsis criteria, the 16% of patients who were discharged from the ED rather than admitted to the hospital were younger, less ill, and more likely to have urinary tract infections. Physicians’ discharge rates varied significantly, and the adjusted 30-day mortality was noninferior and lower among discharged patients vs admitted patients.

**Meaning:**

Findings of this study suggest that outpatient management of sepsis in patients who present to the ED is more common than previously recognized but is not associated with higher mortality compared with hospital admission.

## Teaser

This cohort study assesses the physician, hospital, and patient factors associated with emergency department discharge or hospital admission of adult patients with clinical sepsis.

## Introduction

Current sepsis treatment guidelines and quality metrics presume that emergency department (ED) patients with sepsis syndrome (defined as organ failure from a dysregulated host response to infection^1^) will be admitted to the hospital for further treatment.^2,3^ Most studies of sepsis to date have focused on patients who were admitted to the hospital (likely more than 1.7 million annually in the US^4^) or the subset of patients who were admitted to an intensive care unit. However, 1 study that applied objective clinical criteria for identifying patients with sepsis in the ED noted incidentally that a substantial fraction of such patients may be discharged from the ED for outpatient treatment.^5^

Unlike for pulmonary embolism and pneumonia,^6,7,8,9,10,11^ validated risk scores, clinical trials, and guidelines are not available to guide outpatient disposition decisions for ED patients with sepsis. To our knowledge, the epidemiology of patients with sepsis who were discharged from the ED has not been previously investigated systematically. Consequently, the extent to which such discharges from the ED of patients with clinical sepsis reflect missed diagnoses, improper treatment judgments, patient preferences, or appropriate triage of low-risk patients to outpatient management is unclear. It is also unclear whether ED sepsis disposition is subject to disparities in patient sex or race and ethnicity or affected by nonclinical factors, such as ED busyness or physician practice style.

In the present study, we therefore investigated the prevalence, risk factors, practice variation, and outcomes for discharge to outpatient treatment among ED patients with sepsis. Specifically, we identified patient, clinician, and system factors that were associated with outpatient disposition and compared risk-adjusted 30-day mortality after the initial inpatient vs outpatient treatment assignment from the ED.

## Methods

This retrospective cohort study was conducted at the EDs of 4 hospitals in central Utah, including 2 community hospitals, a regional referral hospital, and an academic tertiary referral center with a level 1 trauma center. Hospital EDs ranged in size from 19 to 57 beds with 22 000 to 89 000 patient visits annually. Standardized care processes for sepsis had been adopted at all hospitals before this study.^12^ A previous study has reported the association of antibiotic timing with mortality in a subset of this ED cohort who were admitted to the hospital.^13^ The Intermountain Healthcare Institutional Review Board approved this study and waived the informed consent requirement according to the 2018 Common Rule. We followed the Strengthening the Reporting of Observational Studies in Epidemiology (STROBE) reporting guideline.^14^

### Patient Population and Data Collection

Adult patients aged 18 years or older who presented to a participating ED from July 1, 2013, to December 31, 2016, were included in the analysis if they met clinical sepsis criteria before ED departure. Data extraction and analysis were performed from 2017 to 2021. Clinical sepsis in the ED was identified according to international consensus criteria^1^ as the combination of known infection or suspected infection (according to the collection of body fluid cultures in the ED plus the administration of at least 1 intravenous antimicrobial or enteric vancomycin hydrochloride, fidaxomicin, or oseltamivir) and acute organ failure (with a Sequential Organ Failure Assessment [SOFA] score ≥2 points higher than baseline value; score range: 0 [best] to 24 [worst] points) (eMethods in the Supplement). We excluded patients with trauma, those who did not have infection suspected in the ED according to medical record review, those who died in the ED, and those who were discharged from the ED against medical advice or to hospice. Only the first eligible encounter for each patient was included in the analysis. Analyses of physician variation were restricted to patients whose ED attending physician provided care to at least 19 other eligible patients.

Development of the study cohort has been previously described.^13^ Patient demographic characteristics and clinical information were obtained from the electronic data warehouse at Intermountain Healthcare. Data on participants’ 30-day mortality was acquired from an established linkage of hospital records to Utah state death records and the US Social Security Death Index. Manual abstraction of each medical record by our team of trained personnel (4 research coordinators and 2 medical students [S.R.M and E.M.]) enabled us to identify patients who were admitted from a long-term care facility; to correct missing data (resulting in no missing data for variables other than white blood cell count); to verify apparent outlying values; and, using standardized a priori criteria, to confirm that infection was suspected by the ED care team and to adjudicate the ED-diagnosed source of infection. Independent review of the ED-diagnosed infection source by a second member of the abstraction team or a physician investigator (I.D.P.) for 31% of the cohort yielded a κ score of 0.96 (95% CI, 0.95-0.97). Infection source abstractions that were flagged for further review by the primary abstractor and all determinations that the patient did not have infection as suspected by the ED physician were reviewed by a physician investigator (I.D.P.). This investigator also resolved between-abstractor disagreements.

### Exposures and Outcomes

We dichotomized ED disposition as hospital admission or discharge to outpatient care from the ED. Patients who were transferred from the ED to another acute care hospital, admitted to the hospital on observation status, or placed on ED observation status were considered to be admitted to the hospital. Patients who were transferred to a psychiatric facility, long-term care facility, or skilled nursing facility were considered discharged from the ED. The primary ED attending physician was identified using a validated algorithm that was supplemented by manual medical record review. Patients' race and ethnicity were based on self-report, observation, or clinical documentation in the electronic medical record and are reported as Hispanic/Latino, non-Hispanic/Latino Black, non-Hispanic/Latino White, or other (including non-Hispanic/Latino Asian, American Indian or Alaska Native, and Native Hawaiian or Other Pacific Islander). Race and ethnicity were dichotomized for multivariable analyses as non-Hispanic/Latino White or other race or Hispanic/Latino ethnicity.

The Mortality in Emergency Department Sepsis (MEDS) score (range, 0 [lowest predicted mortality] to 27 [highest predicted mortality]) and the weighted Elixhauser Comorbidity Index score, which was derived by the von Walraven modification, were calculated as previously described.^15,16^ The ED occupancy rate was defined as the ratio of registered patients to licensed ED beds.^17,18^ Additional information on definitions and validation for study outcomes, exposures, and other data elements is provided in the eMethods in the Supplement, including derivation of variables to characterize the trajectory of vital sign parameters during the ED visit.

### Statistical Analysis

For descriptive comparisons, we used χ^2^ for categorical variables or an unpaired, 2-tailed *t* test with unequal variance for continuous variables as appropriate. To analyze the patient and system factors associated with ED disposition, we used penalized logistic regression with LASSO (least absolute shrinkage and selection operator) to select the factors associated with ED discharge from a prespecified list of candidate risk factors and to estimate the magnitude of the association.^19,20^ The eMethods and eFigure 1 in the Supplement provide additional details.

We measured physician variation in ED disposition using generalized linear mixed models that incorporated a random effect for ED attending physician, a logit link, and binomial distribution with adjustment for a prespecified list of potential confounders. These confounders were nighttime arrival; weekend arrival; MEDS score; triage acuity score (Canadian Triage and Acuity Scale); age; sex; hospital; race and ethnicity; English as preferred language; mode of arrival to ED; abnormal initial Glasgow Coma Scale score; and initial systolic blood pressure, temperature, and heart rate. To test significance, we used a likelihood ratio test that compared the mixed model with a model that excluded the random effect for physician.

In the primary analysis, we applied inverse probability of treatment weighting (IPTW) based on a propensity score,^21,22^ incorporating the LASSO-identified discharge risk factors (C statistic, 0.881; 95% CI, 0.876-0.890) to evaluate whether patients with sepsis who were discharged from the ED experienced noninferior^23^ risk-adjusted 30-day mortality. In the prespecified sensitivity analyses, we used alternative analytic frameworks: (1) matching based on the propensity score, (2) inverse probability weighting with regression adjustment,^24,25^ (3) propensity score adjustment, and (4) multivariable logistic regression. Both IPTW and propensity matching yielded generally well-balanced groups (eFigure 2 in the Supplement); additional details are provided in the eMethods in the Supplement. However, to evaluate potential bias from imperfect propensity score overlap, we also performed a post hoc sensitivity analysis that repeated the primary IPTW analysis after excluding patients with more extreme propensity for either treatment assignment (ie, propensity score <0.1 or >0.9).^26,27^

Informed by a review of the literature^7,10,28,29^ and expert input, we set a noninferiority boundary for 30-day mortality as a +1.5% absolute risk difference after conversion to an odds ratio (OR) and tested it with a 1-sided α = .05. For all other analyses, including superiority analyses of the mortality outcome, a 2-sided *P* ≤ .05 was considered to be statistically significant. Analyses were performed using Stata, version 16.1 (StataCorp LLC) and R, version 4.1.0 (R Foundation for Statistical Computing).

## Results

The 12 333 patients who met the international sepsis criteria while in the ED had a median (IQR) age of 62 (47-76) years and consisted of 7017 women (56.9%) and 5316 men (43.1%). In total, 1985 patients (16.1%) were discharged from the ED for outpatient care rather than admitted to the hospital (eFigure 3 in the Supplement). Discharged patients were younger; were more often female; were less likely to arrive by ambulance or from a long-term care facility; and had less comorbid illness, lower illness acuity, and less organ dysfunction (Table 1). Urinary tract infections were substantially more common among discharged patients (1313 [66.2%]) compared with admitted patients (2733 [26.4%]), and discharged patients were less likely than admitted patients to have pneumonia (195 [9.8%] vs 3436 [33.2%]) or intra-abdominal infection (42 [2.1%] vs 1025 [9.9%]).

**Table 1.** Characteristics of Emergency Department Patients With Sepsis by Discharge Disposition

Patient characteristic^a^	No. (%)		*P* value
	Admitted to the hospital (n = 10 348)	Discharged from the ED (n = 1985)	
Age, mean (SD), y	61.5 (18.9)	54.1 (20.8)	<.001
Sex			
Male	4652 (45.0)	664 (33.5)	<.001
Female	5696 (55.0)	1321 (66.5)	
Race and ethnicity^b^			
Hispanic/Latino	956 (9.2)	264 (13.3)	<.001
Non-Hispanic/Latino Black	131 (1.3)	33 (1.6)	
Non-Hispanic/Latino White	8674 (83.8)	1582 (79.7)	
Other^c^	587 (5.7)	106 (5.3)	
English as preferred language	9788 (94.6)	1875 (94.5)	.82
Currently married	5288 (51.1)	1043 (52.5)	.24
Primary insurance			
Private	2892 (28.0)	767 (38.6)	<.001
Medicare	5479 (53.0)	768 (38.7)	
Medicaid	975 (9.4)	1644 (8.3)	
Uninsured	1002 (9.7)	286 (14.4)	
Arrival to ED by ambulance	3066 (29.6)	238 (12.0)	<.001
Arrival to ED from long-term care facility	681 (6.6)	54 (2.7)	<.001
ED occupancy rate, mean (SD)	0.65 (0.29)	0.65 (0.29)	.86
ED arrival			
Nighttime	1178 (11.4)	234 (11.8)	.60
Weekend	2828 (28.3)	567 (28.6)	.26
MEDS score	6.7 (3.6)	4.4 (3.2)	<.001
SOFA score	4.5 (2.6)	2.6 (0.9)	<.001
Weighted Elixhauser Comorbidity Index score	0 (0-3)	0 (0-0)	<.001
CTAS score: emergent or resuscitation	5504 (52.9)	559 (28.2)	<.001
Initial ED vital signs			
Temperature, mean (SD), °C	37.4 (1.3)	37.3 (1.1)	<.001
Respiratory rate	20 (18-23)	18 (16-20)	<.001
Systolic blood pressure, mm Hg	130 (27)	131 (23)	.04
Heart rate	102 (23)	95 (21)	<.001
Glasgow Coma Scale score ≤13	538 (5.2)	23 (1.2)	<.001
Initial ED laboratory results			
Lactate measured and >2 mmol/L	3700 (35.5)	229 (11.5)	<.001
WBC count, mean (SD), 1000/dL^a^	13.2 (13.1)	10.5 (4.4)	<.001
Source of infection diagnosed in ED			
Pneumonia	3436 (33.2)	195 (9.8)	<.001
Urinary tract	2733 (26.4)	1313 (66.2)	
Intra-abdominal	1025 (9.9)	42 (2.1)	
Skin	973 (9.4)	149 (7.5)	
Other	2181 (21.1)	286 (14.4)	
ED length of stay, min	289 (109)	303 (127)	<.001
30-d Mortality	854 (8.3)	18 (0.9)	<.001

Abbreviations: CTAS, Canadian Triage and Acuity Scale (score range: 1 [resuscitation] to 5 [nonurgent]); ED, emergency department; MEDS, Mortality in Emergency Department Sepsis (score range: 0 [lowest predicted mortality] to 27 [highest predicted mortality]); SOFA, Sequential Organ Failure Assessment (score range: 0 [best] to 24 [worst]); WBC, white blood cell.

^a^ No missing data for variables other than initial WBC count (n = 22).

^b^ Race and ethnicity were based on self-report, observation, or clinical documentation in the electronic medical record.

^c^ Other race and ethnicity included non-Hispanic/Latino Asian, American Indian or Alaska Native, and Native Hawaiian or Other Pacific Islander.

A blood culture was collected in the ED for most patients (8715 [70.7%]), of whom 612 (7.0%) were discharged from the ED. Based on their MEDS score, 55% of discharged patients (n = 1088) and 26% of admitted patients (n = 2656) were in the lowest risk category, with a predicted 28-day mortality of 1.1% (eTable in the Supplement). Conversely, 17% of discharged patients (n = 343) and 42% of admitted patients (n = 4298) had a MEDS score predicting 28-day mortality of 9.3% or higher. The SOFA score increases were larger among admitted patients with sepsis vs discharged patients for all 6 component organ systems, with the respiratory system component exhibiting the largest absolute difference (1.50 vs 0.86; *P* < .001) and the central nervous system component exhibiting the largest relative difference (0.37 vs 0.01; *P* < .001) (eFigure 4 in the Supplement).

Variables that were identified as informative regarding the ED disposition of patients with sepsis using penalized regression are shown in Table 2. Demographic characteristics that retained a significant association with ED discharge included age (adjusted OR [aOR], 0.90 per 10-year increase; 95% CI, 0.87-0.93), arrival by ambulance (aOR, 0.61; 95% CI, 0.52-0.71), comorbidity score (aOR, 0.94; 95% CI, 0.94-0.96), and organ failure severity (aOR, 0.58 per 1-point increase in the Sequential Organ Failure Assessment score; 95% CI, 0.54-0.60). As shown in Table 2, abnormal lactate and vital sign were also associated with ED discharge. Compared with patients with pneumonia, patients with abdominal infections were less likely to be discharged from the ED (aOR, 0.51; 95% CI, 0.39-0.65), whereas patients with urinary tract (aOR, 4.56; 95% CI, 3.91-5.31), skin (aOR, 1.40; 95% CI, 1.14-1.72), and other (aOR, 1.67; 95% CI, 1.40-1.97) infections were more likely to be discharged from the ED. In contrast to the unadjusted analysis, in the adjusted analysis, female sex was not significantly associated with a lower likelihood of ED discharge (aOR, 0.90; 95% CI, 0.80-1.00). The ED occupancy rate and patient arrival time were among the potential risk factors that were not identified as informative (Table 2).

**Table 2.** Factors Associated With Discharge Rather Than Admission of Emergency Department Patients With Sepsis

Candidate risk factor selected by LASSO^a^	Adjusted OR (95% CI)
Age, per 10-y increase	0.90 (0.87-0.93)
Sex	
Female	0.90 (0.80-1.00)
Male	1 [Reference]
Race and ethnicity^b^	
Non-Hispanic/Latino White	1 [Reference]
Other race or Hispanic/Latino ethnicity	1.01 (1.00-1.17)
Currently married	1.00 (1.00-1.12)
Primary insurance	
Private	1 [Reference]
Medicare	0.96 (0.85-1.00)
Medicaid	0.95 (0.80-1.00)
Uninsured	1.01 (0.95-1.12)
Arrival to ED by ambulance	0.61 (0.52-0.71)
Arrival to ED from long-term care facility	0.94 (0.70-1.00)
Weighted Elixhauser Comorbidity Index score	0.94 (0.94-0.96)
SOFA score	0.58 (0.54-0.60)
CTAS score, level	
Resuscitation	1 [Reference]
Emergent	0.92 (0.70-1.10)
Urgent	1.04 (0.84-1.32)
Semiurgent or nonurgent	0.96 (0.70-1.30)
Initial ED vital signs	
Temperature, °C	1.04 (1.00-1.10)
Respiratory rate	0.98 (0.97-1.00)
Initial oxygen saturation	1.00 (1.00-1.01)
Systolic blood pressure, per 10-mm Hg increase	0.92 (0.88-0.94)
Change in vital signs	
Heart rate, per 10-bpm increase	0.82 (0.79-0.84)
Systolic blood pressure, per 10-mm Hg increase	1.12 (1.08-1.15)
Systolic blood pressure trajectory in ED^c^	
Initially normal/deteriorating	1.00 (0.92-1.12)
Initially abnormal/improving	0.93 (0.76-1.00)
Persistently abnormal	1.09 (1.00-1.44)
Transiently abnormal	1.01 (0.98-1.08)
Respiratory rate trajectory in ED^c^	
Initially normal/deteriorating	1.12 (0.94-1.34)
Initially abnormal/improving	0.71 (0.62-0.81)
Persistently abnormal	0.92 (0.76-1.12)
Transiently abnormal	0.70 (0.62-0.78)
Heart rate trajectory in ED^c^	
Initially normal/deteriorating	0.46 (0.32-0.62)
Initially abnormal/improving	0.84 (0.76-0.92)
Persistently abnormal	0.95 (0.82-1.10)
Transiently abnormal	0.58 (0.48-0.70)
Lactate measured and >2 mmol/L	0.62 (0.53-0.72)
Source of infection diagnosed in ED	
Pneumonia	1 [Reference]
Urinary tract	4.56 (3.91-5.31)
Intra-abdominal	0.51 (0.39-0.65)
Skin	1.40 (1.14-1.72)
Other	1.67 (1.40-1.97)

Abbreviations: CTAS, Canadian Triage and Acuity Scale (score range: 1 [resuscitation] to 5 [nonurgent]); ED, emergency department; LASSO, least absolute shrinkage and selection operator; OR, odds ratio; SOFA, Sequential Organ Failure Assessment (score range: 0 [best] to 24 [worst]).

^a^ Variables that were not selected by LASSO included ED occupancy rate, Mortality in Emergency Department Sepsis score, preferred language, initial ED heart rate and Glasgow Coma Scale score, respiratory rate change, and arrival time.

^b^ Race and ethnicity were based on self-report, observation, or clinical documentation in the electronic medical record. Other race included non-Hispanic/Latino Asian, American Indian or Alaska Native, Black, and Native Hawaiian or Other Pacific Islander.

^c^ The reference category when assessing overall vital sign trajectories was the persistently normal group.

Analysis of physician variation included 12 258 patients who were treated by 89 ED attending physicians (Table 3) who provided care to at least 20 eligible patients with sepsis. The median (IQR) number of patients per physician was 137 (109-180), and the unadjusted physician-level discharge rates ranged from 0% to 39%. After adjustment for patient demographic and clinical characteristics, ED attending physician was significantly associated with discharge probability (likelihood ratio test, *P* < .001), with physician-level discharge rates ranging from 8% to 40% for an average patient (Figure).

**Table 3.** Demographic Characteristics of Emergency Department Physicians

Clinician characteristic	Physicians, No. (%) (n = 89)
Age, median (IQR), y	39 (35-48)
Sex	
Female	17 (19.1)
Male	72 (80.9)
Years of experience after residency, median (IQR)	8 (4-18)
Emergency medicine residency^a^	75 (84.3)
Emergency medicine board certification	78 (87.6)

^a^ Included combined residencies.

**Figure.**
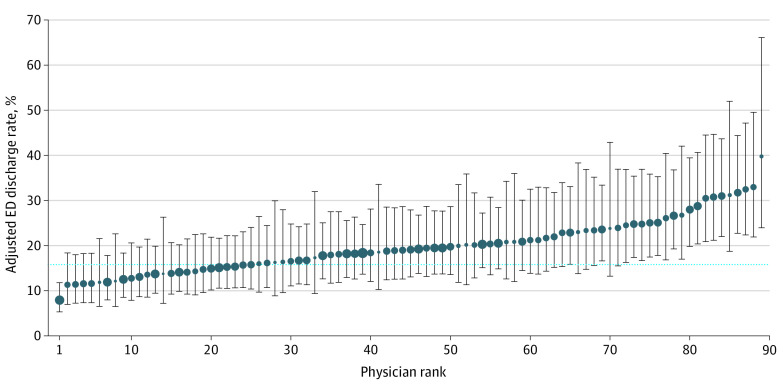
Variation in the Adjusted Probability of Discharge by Emergency Department (ED) Attending Physician Point estimates (with 95% CIs) represent the physician’s estimated probability of discharging a patient along with the population’s mean adjusted discharge probability (15.8%, dotted line). Marker sizes are proportional to the number of cases seen by each physician.

The unadjusted 30-day mortality was 8.3% among patients with sepsis who were admitted to the hospital compared with 0.9% for patients who were discharged from the ED (OR, 0.10; 95% CI, 0.06-0.16). After stratification according to MEDS-based mortality risk, 30-day mortality remained lower among discharged patients across risk groups (eTable in the Supplement). After accounting for the propensity for discharge from the ED, the risk of dying within 30 days of ED arrival was 1.7% (95% CI, 1.4%-2.1%) among patients who were discharged from the ED compared with 7.5% (95% CI, 6.8%-8.1%) among patients who were admitted to the hospital (risk difference, 5.8%; 95% CI, 5.1%-6.5%; *P* < .001). Compared with admitted patients, discharged patients had a propensity-adjusted 30-day mortality that was noninferior and significantly lower (aOR, 0.21; 95% CI, 0.09-0.48). Sensitivity analyses that used alterative statistical methods to control for confounding factors yielded aOR point estimates that ranged from 0.21 to 0.42 (Table 4).

**Table 4.** Primary and Sensitivity Analyses of Adjusted Association of 30-Day Mortality With Discharge Rather Than Hospital Admission for ED Patients With Sepsis

Analysis method	OR for 30-d mortality for patients discharged from ED (95% CI)	*P* value
Unadjusted analysis	0.10 (0.06-0.16)	<.001
Propensity-based IPTW^a^	0.21 (0.09-0.48)	<.001
Propensity score matching^a^^,^^b^	0.35 (0.20-0.61)	<.001
Propensity-based IPTW with regression adjustment^a^^,^^c^	0.28 (0.22-0.35)	<.001
Propensity score adjustment	0.42 (0.22-0.72)	.004
Multivariable logistic regression^c^	0.25 (0.15-0.39)	<.001
Propensity-based IPTW after trimming extreme propensity scores^a^^,^^d^	0.21 (0.18-0.61)	<.001

Abbreviations: ED, emergency department; IPTW, inverse probability of treatment weighting; OR, odds ratio.

^a^ Estimated average treatment effect in study population.

^b^ Included 1653 matched pairs and 3306 unique patients.

^c^ Adjusted for age, sex, comorbidity index score, mode of arrival to ED, residence in a nursing home or long-term care facility, nighttime ED arrival, pooled triage acuity score, systolic blood pressure trajectory category, abnormal initial Glasgow Coma Scale score, and ED-diagnosed source of infection.

^d^ Included 5714 patients with a propensity score in the range from 0.1 to 0.9.

## Discussion

In a multicenter cohort study of ED patients with clinical sepsis, we found that 16.1% of patients were discharged from the ED. There was substantial practice variation in discharge disposition among physicians. Even after comprehensive risk adjustment, the 30-day mortality was noninferior and was significantly lower among discharged patients compared with admitted patients, with OR point estimates for the association of ED discharge with mortality ranging from 0.21 to 0.42. We hypothesize that this finding indicates that many ED clinicians are able to synthesize objective and subjective information to identify a subset of patients with sepsis who may safely be treated in the outpatient setting.

The findings were consistent with previous preliminary data that suggested the setting for sepsis care was less uniform than commonly assumed.^5,30^ In 1 study, 13% percent of ED patients who were diagnosed with septicemia or disseminated infection were not admitted to the hospital, including 2.3% of patients who were explicitly diagnosed with both infection and organ failure (ie, sepsis).^30^ That study identified patients with sepsis using insensitive administrative methods (discharge diagnosis codes), which tend to capture only the 50% to 70% of patients with sepsis who are more severely ill.^4,31,32^ Consequently, that previous study likely substantially underestimated the true rate of outpatient sepsis management by excluding less ill patients with sepsis who were more likely to be discharged from the ED.^30^ By contrast, another study that used clinical criteria to identify sepsis among ED patients incidentally noted that 20% to 25% of such patients were not admitted to the hospital.^5^ Similar to its predecessor, however, the study focused on measuring the prevalence of sepsis among ED patients and provided no data on the characteristics or outcomes of the patients who were discharged from the ED.^5^

The present study provides a granular characterization of discharged ED patients with sepsis and suggests that these patients’ ED disposition largely, but not entirely, reflects appropriate triage of low-risk patients to outpatient care rather than missed diagnoses or improper disposition judgments. Most discharged patients had low predicted mortality, and the patient factors associated with discharge decision were restricted to age, indicators of illness severity (including organ failure severity in particular), and infection source rather than nonclinical factors, such as sex, race and ethnicity, or ED busyness. Furthermore, the finding that 30-day mortality among patients with sepsis was lower than in admitted patients suggested that some patients with acute organ failure and both suspected and confirmed infection may be safely treated in the outpatient setting.

Appropriate triage to outpatient management will not be synonymous with perfect outcomes; the observed 30-day sepsis mortality of 0.9% in the present cohort was actually somewhat lower than the expected mortality among ED patients with pneumonia and pulmonary embolism who are considered appropriate for outpatient care.^6,7,8,9,10,11^ In addition, we note that benchmarking using the outcomes of only admitted patients with sepsis may lead to substantial variation in reported outcomes that is independent of the quality of care but affected by the rates of admission of patients who are suitable candidates for outpatient care.

### Strengths and Limitations

This study has several strengths. The study analyzed a large, multihospital cohort of well-characterized ED patients, used robust statistical methods, and had the ability to capture 30-day mortality among patients who were discharged from the ED. However, we emphasize that the findings do not currently support interventions to increase the fraction of ED patients with sepsis who are discharged from the ED to outpatient treatment. The findings do suggest a need for an improved evidence base for ED disposition and provide data that are relevant to eventual randomized clinical trials. The observed significant, between-physician variation in risk-adjusted discharge rates indicates that physician practice habits rather than patient preferences or clinical factors are an important contributor to patient disposition decision and suggests an opportunity for further research to help physicians make evidence-based triage decisions. Clinical decision support tools that are similar to those validated for guiding care location for ED patients with community-acquired pneumonia or pulmonary embolism may be warranted to optimize both the undertriage and overtriage of patients with sepsis.^6,7,8,9,10,11,33,34^ However, the utility of such tools could be impaired and the risk of harm could be increased by the challenging nature of bedside sepsis identification, including the substantial underdiagnosis of lower-severity sepsis cases.^2,4,35,36^

This study also has several limitations. The most important of these limitations is the likelihood, despite adjustment for multiple potential confounders, of indication bias and unmeasured confounding in the mortality analysis, as suggested by the statistically lower mortality in patients who were discharged to outpatient treatment. The adjusted estimates for the association of ED discharge with mortality ranged from 0.21 to 0.42, with smaller effect sizes obtained with more effective adjustment strategies, including propensity matching (which yielded better covariate balance) and regression-adjusted IPTW (which is more robust to model specification) compared with the primary IPTW analysis.^25,37^ The mortality findings should, therefore, not be interpreted as evidence that outpatient management is associated with reduced mortality for patients with sepsis. We were unable to evaluate health care utilization after ED discharge, including follow-up with primary care or specialty physicians, planned or unplanned return visits to urgent care clinics or the ED, and subsequent hospital admissions. The availability of short-term follow-up via outpatient clinicians or a planned recheck in the ED may be a key factor in many ED disposition decisions. Although some markers of socioeconomic status and social support, including insurance type and marital status, were not associated with disposition in the adjusted analysis, we were unable to evaluate other potentially important factors associated with follow-up reliability specifically and the disposition decision generally, such as substance use, other measures of socioeconomic status, and homelessness.^38^ Other unassessed factors potentially affecting disposition include whether the ED clinician explicitly diagnosed sepsis (which could increase the likelihood of admission) and ED clinicians’ degree of certainty regarding the presence of infection. These factors should be investigated in future quantitative studies of post-ED health care utilization among patients with sepsis who were discharged from the ED and qualitative studies of ED clinicians’ decision-making around ED disposition.

Other study limitations include the lack of racial and ethnic diversity and sex imbalance among included ED physicians, which is typical of the demographic characteristics of US emergency medicine physicians.^39^ Some between-group standardized mean differences for covariates remained larger than optimal after IPTW,^40^ but results from the well-balanced, propensity-matched sensitivity analysis of mortality were reassuring regarding the validity of the primary IPTW analysis. Incomplete data used in the calculation of baseline SOFA may have led to overestimation of acute organ failure, and concurrent acute illnesses other than patients’ suspected infections may have been a factor in acute organ failure events. Some patients who were diagnosed with and treated for infection in the ED likely had noninfectious final diagnoses.^41^

## Conclusions

This cohort study found that ED discharge was associated with noninferior and significantly lower mortality at 30 days among the 16.1% of ED patients with clinical sepsis who were discharged from the ED compared with patients who were admitted to the hospital. The findings suggest that outpatient treatment of patients with sepsis is more common than previously recognized but is not associated with higher mortality than hospital admission. Age, infection source, and measures of illness severity and organ failure were associated with the probability of ED discharge of patients with sepsis, but substantial physician-level variation in risk-adjusted discharge rates was also observed. Systematic, evidence-based strategies to optimize the triage of ED patients with sepsis are needed.

## Notes

Supplement. **eMethods.** Supplementary Methods **eFigure 1.** Cross Validation Error for Each Candidate Model **eFigure 2.** Comparison of Standardized Mean Differences and Variance Ratios for the Unweighted Sample After Inverse Probability of Treatment Weighting (IPTW) or Propensity Matching **eFigure 3.** Patient Inclusion/Exclusion Diagram **eFigure 4.** Emergency Department Sequential Organ Failure Assessment (SOFA) Component Score Increase by Emergency Department Disposition **eTable.** Observed Versus Predicted Mortality by ED Disposition
eReferences
