# Supplementary material for: Prevalence, Characteristics, and Outcomes of Emergency Department Discharge Among Patients With Sepsis
Source: JAMA Netw Open. 2022 Feb 10;5(2):e2147882. doi: 10.1001/jamanetworkopen.2021.47882 (PMC8832179; doi:10.1001/jamanetworkopen.2021.47882)
Supplement: Supplement. — eMethods. Supplementary Methods eFigure 1. Cross Validation Error for Each Candidate Model eFigure 2. Comparison of Standardized Mean Differences and Variance Ratios for the Unweighted Sample After Inverse Probability of Treatment Weighting (IPTW) or Propensity Matching eFigure 3. Patient Inclusion/Exclusion Diagram eFigure 4. Emergency Department Sequential Organ Failure Assessment (SOFA) Component Score Increase by Emergency Department Disposition eTable. Observed Versus Predicted Mortality by ED Disposition eReferences [file jamanetwopen-e2147882-s001.pdf]

Supplementary Online Content

Peltan ID, McLean SR, Murnin E, et al. Prevalence, characteristics, and outcomes of emergency department discharge among patients with sepsis. *JAMA Netw Open*. 2022;5(2):e2147882. doi:10.1001/jamanetworkopen.2021.47882

eMethods. Supplementary Methods

eFigure 1. Cross Validation Error for Each Candidate Model

eFigure 2. Comparison of Standardized Mean Differences and Variance Ratios for the Unweighted Sample After Inverse Probability of Treatment Weighting (IPTW) or Propensity Matching

eFigure 3. Patient Inclusion/Exclusion Diagram

eFigure 4. Emergency Department Sequential Organ Failure Assessment (SOFA) Component Score Increase by Emergency Department Disposition

eTable. Observed Versus Predicted Mortality by ED Disposition

eReferences

This supplementary material has been provided by the authors to give readers additional information about their work.

© 2022 Peltan ID et al. *JAMA Network Open.*

eMethods. Supplementary Methods

Validation of emergency department (ED) disposition

Primary data on ED disposition location was compared to admission status, discharge transport mode, ED discharge destination, and hospital length of stay and verified by manual chart review if potential discordance was observed.

Sequential Organ Failure Assessment (SOFA) score calculation

The SOFA score was calculated as previously described.[1] Acute SOFA values were obtained from data available between ED arrival and ED departure. The baseline SOFA score was calculated using the most recent data available between 24 hours and 3 years prior to the index ED arrival (ignoring urine output). When an arterial partial pressure of oxygen (P_aO_2) was unavailable, P_aO_2 was imputed from the peripheral oxygen saturation using a non-linear equation.[2] Otherwise, in accordance with the Sepsis-3 guidelines, prior studies and clinical practice,[3-5] acute and baseline SOFA component scores were assumed to be 0 if data for that component were unavailable. Among 12,333 patients, 4,175 (34%) had objective data available for 1 or more components of the baseline SOFA score.

Identification of primary ED attending physician

An automated algorithm that compared (1) authorship of all ED physician notes, (2) attending physician from ED patient tracking "dashboards;" and (3) ED billing clinician assigned a primary ED attending physician for 98.7% of records. For the remaining records, the primary ED attending physician was identified by manual chart review. Comparison of 1,151 records to data abstracted manually for an independent sepsis registry by trained sepsis registrar nurses demonstrated agreement for 99.7% of algorithmic physician assignments.

Vital sign trajectory

In addition to recording subjects' first measured vital signs in the ED, we derived variables for the change in the heart rate, systolic blood pressure, and respiratory rate from first to final ED measurement. We also created variables that categorized the trajectory of each of these three vital signs during the ED stay into five categories: persistently normal, persistently abnormal, initially normal and deteriorating, initially abnormal and improving, and transiently abnormal. Thresholds for abnormal were systolic blood pressure < 100 mmHg, respiratory rate ≥22 breaths per minute, and heart rate >100 beats per minute.

Other variable definitions

- Triage acuity: Triage acuity category recorded in the medical record was assigned by ED nurses using the 5-point Canadian Triage and Acuity Scale.[6] The lowest category (non-urgent) was rarely used and was combined with the next lowest category (semi-urgent) for analyses
- Source of arrival to ED: skilled nursing or long-term care facility versus non-clinical setting.
- Insurance: categorized as private, Medicare, Medicaid, or uninsured/none.
- Marital status: dichotomized as unmarried (including currently separated) versus unmarried
- Preferred language: dichotomized as English versus other
- Mode of arrival to ED: dichotomized as ambulance versus walk-in
- Nighttime ED arrival: defined as midnight to 6:59 AM
- Weekend ED arrival: defined as 12:00 am on Saturday morning to 11:59 pm on Sunday evening
- Abnormal initial Glasgow Coma Scale (GCS) score: defined as GCS ≤13

Factors associated with ED discharge

We employed penalized logistic regression using Least Absolute Shrinkage and Selection Operator (LASSO) to select parameters associated with sepsis patients' ED disposition from a prespecified list of candidate variables, which included patient characteristics (age, sex, race/ethnicity, marital status, preferred language, insurance type, long-term care facility residence), clinical information available while in the ED (arrival to ED via ambulance; comorbidity score; SOFA score; triage acuity; Mortality in ED Sepsis score; initial ED vital signs [temperature, respiratory rate, oxygen saturation, heart rate, GCS, systolic blood pressure]; absolute change in respiratory rate, heart rate, and systolic blood pressure; systolic blood pressure, respiratory rate, and heart rate trajectory; lactate; infection source), and parameters influencing ED care delivery (ED occupancy rate, ED arrival time). To minimize instability in variable selection and estimation, we ensured a ratio outcome events to candidate risk factors substantially greater than 10:1.[7,8] LASSO reduces model overfitting by reducing the coefficient associated with each variable based on a penalty term (λ), the magnitude of which increases as model complexity increases.[9,10] Variables

© 2022 Peltan ID et al. *JAMA Network Open.*

with coefficients reduced to 0 after penalization are removed from the model. Individual levels of categorical variables were included or excluded from the final model as a group. We employed 10-fold cross validation to identify the λ associated with the minimum cross-validation error and then applied the one standard deviation rule to aid selection of a parsimonious model.[11,12] To account for clustering, a fixed effect for hospital was forced into the model. Penalized coefficients for variables remaining in the model are reported along with bootstrapped 95% confidence intervals.

Analysis of 30-day mortality
We recognized that the association of ED disposition and mortality is subject to confounding by indication.[13] The primary analysis employed inverse probability of treatment weighting (IPTW) based on a propensity score created using variables identified as associated with ED disposition as described above.[14-16] eFigure 2 demonstrates improved demographic and clinical parameter balance after IPTW. Values reported represent the average treatment effect (ATE).

We performed several sensitivity analyses using alternative methods to account for indication bias and confounding:

1. Propensity matching: we matched discharged and admitted patients 1:1 based on the propensity score using a greedy matching with replacement and a caliper radius of 0.2 of the pooled standard deviation of the logit of the propensity score. This yielded 1653 matched pairs comprised of 3306 unique patients. As with IPTW, matched pairs exhibited improved demographic and clinical parameter balance (eFigure 2). To account for matching in the analysis, we employed conditional logistic regression to generate effect estimates.
2. Multivariable logistic regression: Conventional multivariable logistic regression adjusting for a prespecified list of potential confounders: age, sex, comorbidity score, mode of arrival to ED, residence in a nursing home or long-term care facility, nighttime ED arrival, pooled triage acuity score, systolic blood pressure trajectory category, abnormal initial Glasgow Coma Scale score, and the ED-diagnosed source of infection.
3. Propensity-based inverse probability of treatment with regression adjustment (IPWRA): IPWRA combines an IPTW model (weighting based on inverse treatment probability calculated from the propensity score) with a model for outcome probability.[17] IPWRA models are "doubly robust," meaning that correct specification of either the treatment (IPW) model or the outcome (regression adjustment) model will yield an accurate effect estimate.[18] The propensity score was generated as above; variables included in the regression adjustment were prespecified and were the same as included in the multivariable logistic regression.
4. Propensity score adjustment: multivariable logistic regression incorporating the exposure and the propensity score as a single adjustment term.
5. IPTW with propensity score restriction (post hoc): to evaluate potential bias due to imperfect propensity score overlap, we repeated the primary IPTW analysis after excluding patients with more extreme propensity for either treatment assignment (i.e., propensity scores <0.1 or >0.9).[19,20]

© 2022 Peltan ID et al. *JAMA Network Open.*

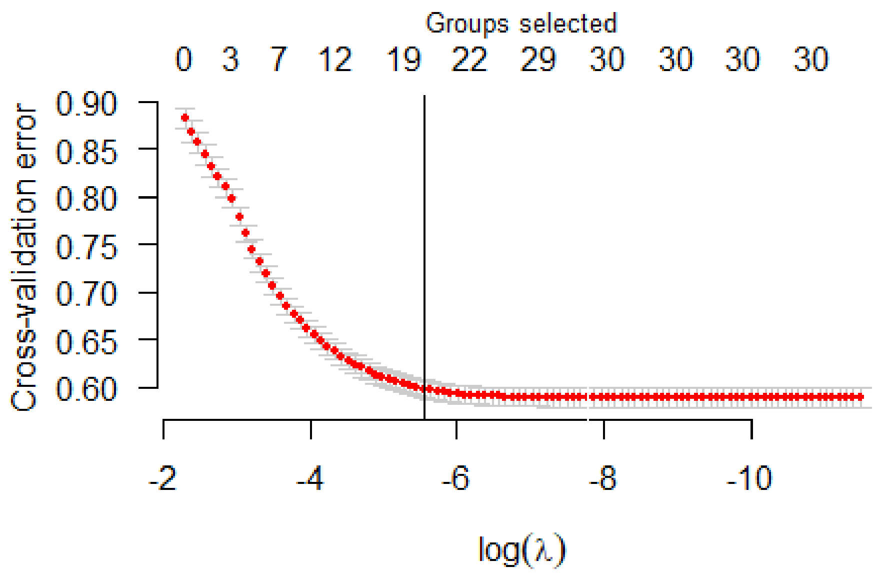

eFigure 1. Cross Validation Error for Each Candidate Model

The solid vertical line indicates the one standard error λ, which is the maximum λ with cross-validation error within one standard deviation of the minimum cross-validation error.

© 2022 Peltan ID et al. *JAMA Network Open.*

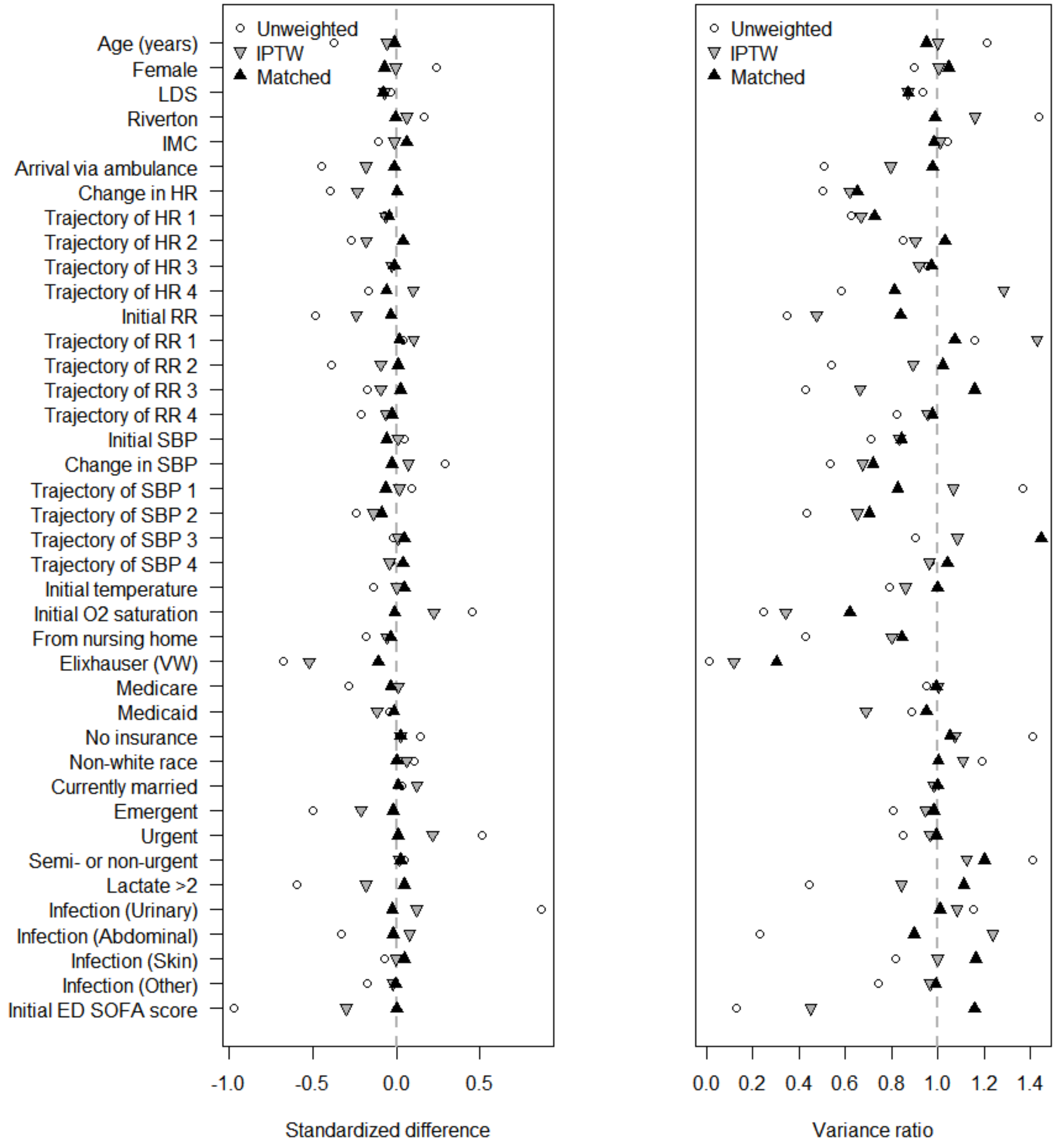

eFigure 2. Comparison of Standardized Mean Differences and Variance Ratios for the Unweighted Sample After Inverse Probability of Treatment Weighting (IPTW) or Propensity Matching

Values closer to 0 for standardized mean differences and values closer to 1 for variance ratio reflect better balance.

© 2022 Peltan ID et al. *JAMA Network Open.*

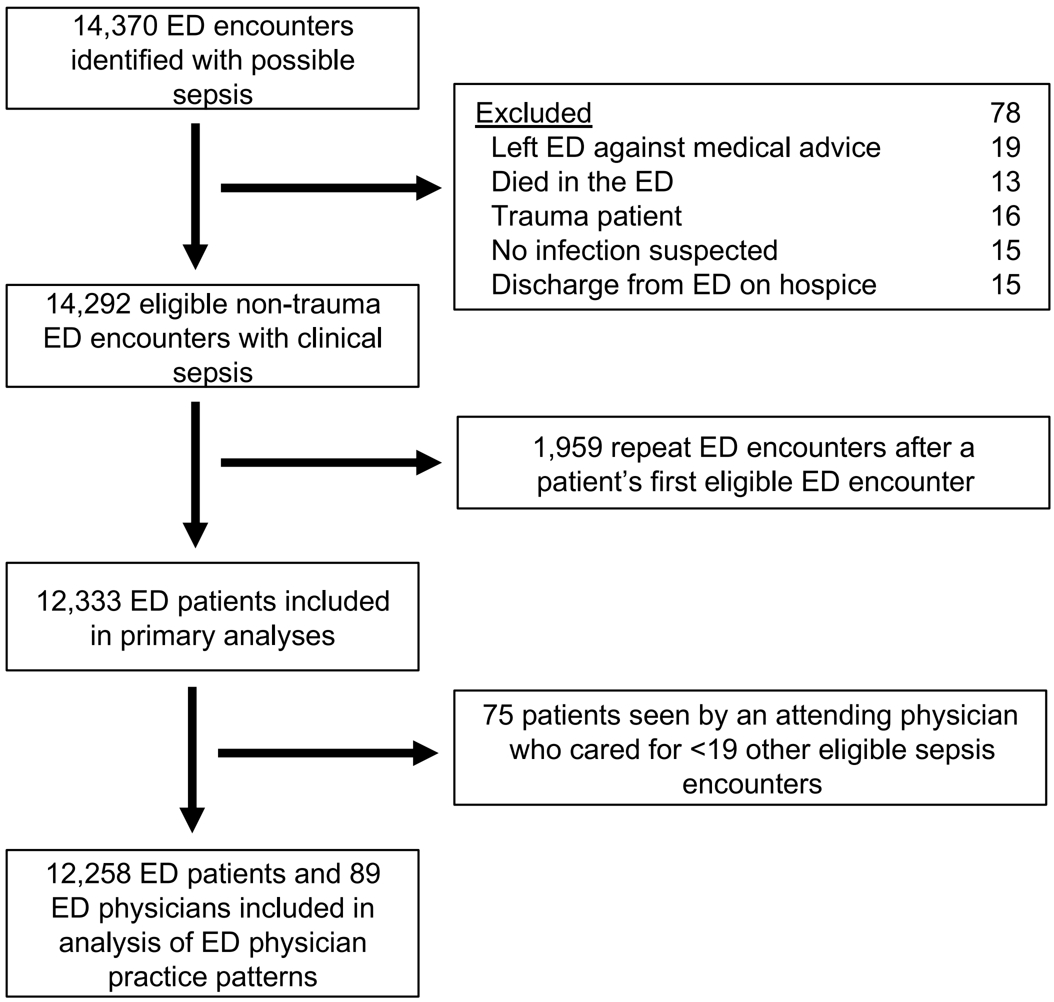

eFigure 3. Patient Inclusion/Exclusion Diagram

© 2022 Peltan ID et al. *JAMA Network Open.*

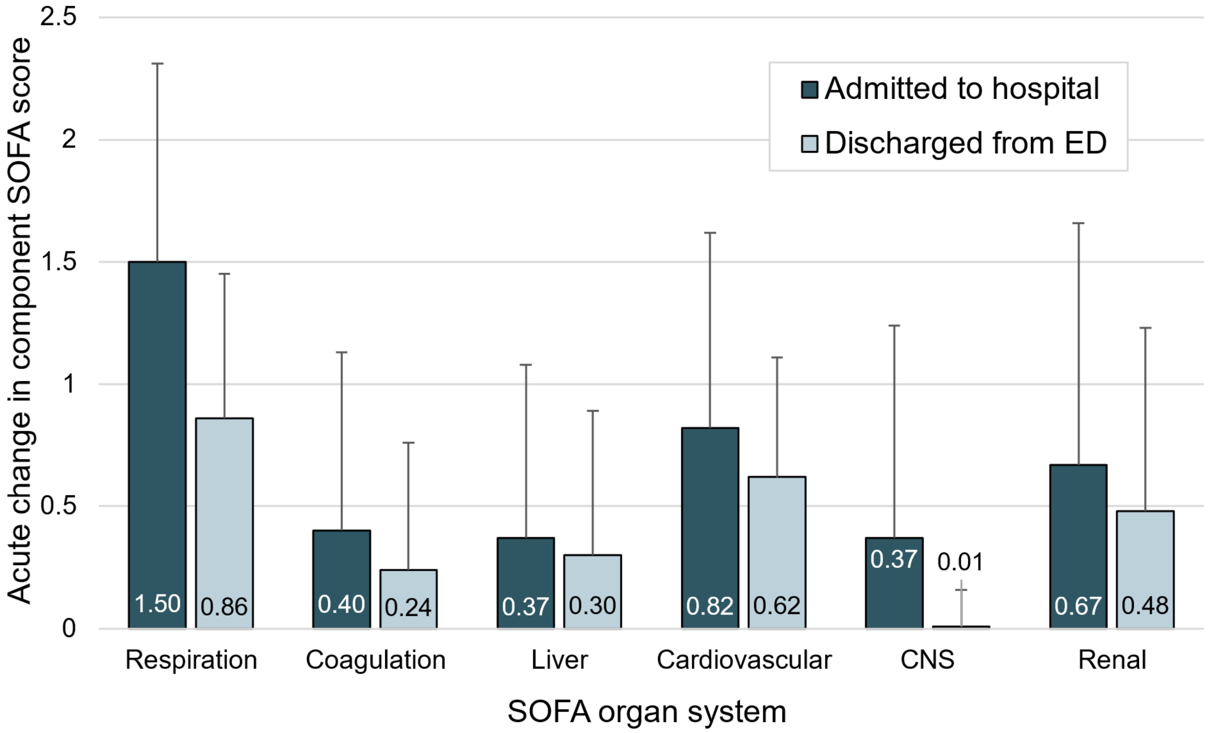

eFigure 4. Emergency Department Sequential Organ Failure Assessment (SOFA) Component Score Increase by Emergency Department Disposition

Values represent change in organ system component score between acute emergency department value and baseline value. p<0.001 for each comparison between hospital admission and discharge from emergency department.
Abbreviations: CNS, central nervous system; ED, emergency department; SOFA, Sequential Organ Failure Assessment score.

© 2022 Peltan ID et al. *JAMA Network Open.*

eTable. Observed Versus Predicted Mortality by ED Disposition

Mortality in ED Sepsis (MEDS) score	Predicted 28-day mortality[a]	Observed 30-day mortality					
		All patients (N=12,333)		Admitted to hospital (N=10,348)		Discharged from ED (N=1,985)	
Score 0-4	1.1%	44/3744	(1.2%)	42/2,656	(1.6%)	2/1088	(0.2%)
Score 5-7	4.4%	163/3948	(4.1%)	156/3,394	(4.6%)	7/554	(1.3%)
Score 8-12	9.3%	450/4024	(11.2%)	442/3,701	(11.9%)	8/323	(2.5%)
Score 13-15	16.1%	141/450	(31.3%)	140/431	(32.5%)	1/19	(5.3%)
Score ≥16	39.1%	74/167	(44.3%)	74/166	(44.6%)	0/1	(0%)

Values reported as mean (standard deviation), N (%) or median (interquartile range).
[a] Predicted 28-day mortality values validation cohort values reported by Shapiro et al.[21]
Abbreviations: ED, emergency department; SOFA, Sequential Organ Failure Assessment

© 2022 Peltan ID et al. *JAMA Network Open.*

eReferences

1. Vincent J-L, Moreno R, Takala J, et al. The SOFA (Sepsis-related Organ Failure Assessment) score to describe organ dysfunction/failure. *Intensive Care Med.* 1996;22(7):707-710.
2. Brown SM, Grissom CK, Moss M, et al. Nonlinear imputation of PaO2/FiO2 from SpO2/FiO2 among patients with acute respiratory distress syndrome. *Chest.* 2016;150(2):307-313.
3. Seymour CW, Liu VX, Iwashyna TJ, et al. Assessment of clinical criteria for sepsis: for the third international consensus definitions for sepsis and septic shock (Sepsis-3). *JAMA.* 2016;315(8):762-774.
4. Singer M, Deutschman CS, Seymour CW, et al. The third international consensus definitions for sepsis and septic shock (Sepsis-3). *JAMA.* 2016;315(8):801-810.
5. Ranzani OT, Prina E, Menendez R, et al. New sepsis definition (Sepsis-3) and community-acquired pneumonia mortality. a validation and clinical decision-making study. *Am J Respir Crit Care Med.* 2017;196(10):1287-1297.
6. Bullard MJ, Unger B, Spence J, Grafstein E, Group CNW. Revisions to the Canadian Emergency Department Triage and Acuity Scale (CTAS) adult guidelines. *CJEM.* 2008;10(2):136-151.
7. Peduzzi P, Concato J, Kemper E, Holford TR, Feinstein AR. A simulation study of the number of events per variable in logistic regression analysis. *J Clin Epidemiol.* 1996;49(12):1373-1379.
8. Wynants L, Bouwmeester W, Moons KGM, et al. A simulation study of sample size demonstrated the importance of the number of events per variable to develop prediction models in clustered data. *J Clin Epidemiol.* 2015;68(12):1406-1414.
9. Efron B, Hastie T, Johnstone I, Tibshirani R. Least angle regression. *Ann Statist.* 2004;32(2):407-499.
10. Zou H, Hastie T. Regularization and variable selection via the elastic net. *J R Statist Soc B.* 2005;67(2):301-320.
11. Breiman L, Friedman JH, Olshen RA, Stone CJ. *Classification and Regression Trees.* Boca Raton: Chapman & Hall/CRC; 1984.
12. Hastie T, Tibshirani R, Friedman JH. *The Elements of Statistical Learning: Data Mining, Inference, and Prediction.* New York: Springer; 2017.
13. Kyriacou DN, Lewis RJ. Confounding by indication in clinical research. *JAMA.* 2016;316(17):1818-1819.
14. Haukoos JS, Lewis RJ. The propensity score. *JAMA.* 2015;314(15):1637-1638.
15. Austin PC. An introduction to propensity score methods for reducing the effects of confounding in observational studies. *Multivariate Behav Res.* 2011;46(3):399-424.
16. Robins JM, Hernán MÁ, Brumback B. Marginal structural models and causal inference in epidemiology. *Epidemiology.* 2000;11(5):550-560.
17. Wooldridge J, Imbens G. Estimation of average treatment effects under unconfoundedness. National Bureau of Economic Research Web site. https://www.nber.org/sites/default/files/2021-03/lect_1_match_fig.pdf. Published 2007. Accessed May 10, 2021.
18. Bang H, Robins JM. Doubly robust estimation in missing data and causal inference models. *Biometrics.* 2005;61(4):962-973.
19. Crump RK, Hotz VJ, Imbens GW, Mitnik OA. Dealing with limited overlap in estimation of average treatment effects. *Biometrika.* 2009;96(1):187-199.
20. Austin PC, Stuart EA. The performance of inverse probability of treatment weighting and full matching on the propensity score in the presence of model misspecification when estimating the effect of treatment on survival outcomes. *Stat Methods Med Res.* 2017;26(4):1654-1670.
21. Shapiro NI, Wolfe RE, Moore RB, Smith E, Burdick E, Bates DW. Mortality in Emergency Department Sepsis (MEDS) score: a prospectively derived and validated clinical prediction rule. *Crit Care Med.* 2003;31(3):670-675.

© 2022 Peltan ID et al. *JAMA Network Open.*
